# A multi-substrate approach for functional metagenomics-based screening for (hemi)cellulases in two wheat straw-degrading microbial consortia unveils novel thermoalkaliphilic enzymes

**DOI:** 10.1186/s12864-016-2404-0

**Published:** 2016-01-28

**Authors:** Mukil Maruthamuthu, Diego Javier Jiménez, Patricia Stevens, Jan Dirk van Elsas

**Affiliations:** Department of Microbial Ecology, Groningen Institute for Evolutionary Life Sciences, University of Groningen, Nijenborgh 7, 9747AG Groningen, The Netherlands

**Keywords:** Metagenomic libraries, Functional screening, Galactosidase, (Hemi)cellulose, Chromogenic substrates, Xylosidase

## Abstract

**Background:**

Functional metagenomics is a promising strategy for the exploration of the biocatalytic potential of microbiomes in order to uncover novel enzymes for industrial processes (e.g. biorefining or bleaching pulp). Most current methodologies used to screen for enzymes involved in plant biomass degradation are based on the use of single substrates. Moreover, highly diverse environments are used as metagenomic sources. However, such methods suffer from low hit rates of positive clones and hence the discovery of novel enzymatic activities from metagenomes has been hampered.

**Results:**

Here, we constructed fosmid libraries from two wheat straw-degrading microbial consortia, denoted RWS (bred on untreated wheat straw) and TWS (bred on heat-treated wheat straw). Approximately 22,000 clones from each library were screened for (hemi)cellulose-degrading enzymes using a multi-chromogenic substrate approach. The screens yielded 71 positive clones for both libraries, giving hit rates of 1:440 and 1:1,047 for RWS and TWS, respectively. Seven clones (NT2-2, T5-5, NT18-17, T4-1, 10BT, NT18-21 and T17-2) were selected for sequence analyses. Their inserts revealed the presence of 18 genes encoding enzymes belonging to twelve different glycosyl hydrolase families (GH2, GH3, GH13, GH17, GH20, GH27, GH32, GH39, GH53, GH58, GH65 and GH109). These encompassed several carbohydrate-active gene clusters traceable mainly to *Klebsiella* related species. Detailed functional analyses showed that clone NT2-2 (containing a beta-galactosidase of ~116 kDa) had highest enzymatic activity at 55 °C and pH 9.0. Additionally, clone T5-5 (containing a beta-xylosidase of ~86 kDa) showed > 90 % of enzymatic activity at 55 °C and pH 10.0.

**Conclusions:**

This study employed a high-throughput method for rapid screening of fosmid metagenomic libraries for (hemi)cellulose-degrading enzymes. The approach, consisting of screens on multi-substrates coupled to further analyses, revealed high hit rates, as compared with recent other studies. Two clones, 10BT and T4-1, required the presence of multiple substrates for detectable activity, indicating a new avenue in library activity screening. Finally, clones NT2-2, T5-5 and NT18-17 were found to encode putative novel thermo-alkaline enzymes, which could represent a starting point for further biotechnological applications.

**Electronic supplementary material:**

The online version of this article (doi:10.1186/s12864-016-2404-0) contains supplementary material, which is available to authorized users.

## Background

Lignocellulose constitutes an abundant organic material that is recalcitrant to degradation. Across different plant species, it contains cellulose (~35–50 %) and hemicellulose (~25–35 %) moieties that are complexed with lignin [1]. The cellulose moiety is a glucose polymer, whereas the hemicellulose part is composed of various pentose and hexose sugars (e.g. xylose, arabinose, mannose and galactose) linked by beta/alpha-glycosidic bonds [2–4]. All of these sugars have great value for the production of bioethanol, biodiesel and/or plastics [5, 6], and so there have been many efforts to release them from the plant matrix. However, current physicochemical methodologies for the degradation of plant biomass and subsequent production of sugars are imperfect [7] and so there is a great interest in the development of alternative and efficient processes, based on enzymes and/or lignocellulolytic microbes [8, 9].

The conversion of plant biomass to sugars requires the concerted action of different proteins, such as carbohydrate-binding modules (CBMs), polysaccharide monooxygenases, pectin lyases, hemicellulases, endoglucanases and beta-glucosidases [10–12]. Among the hemicellulases, xylosidases that can work efficiently at high temperatures in alkaline conditions are highly valued with respect to their usefulness in the pulp bleaching process [13, 14]. Actually, hemicellulases, which have previously been regarded as “accessory enzymes” of cellulases, may themselves exert vital roles in plant biomass hydrolysis [15, 16]. Given the complexity of the enzymes required for efficient lignocellulose breakdown, multi-species microbial consortia offer interesting perspectives [17–20]. To unlock the biocatalytic potential present in lignocellulolytic microbial consortia, metagenomics-based approaches have been proposed [21–23]. Two different strategies can be used: *i*) the unleashing of high-throughput DNA sequencing on degradative consortia, and/or *ii*) the selection of enzymes via functional/genetic screening of metagenomic libraries produced from these consortia [9].

Functional metagenomic screening includes the detection of “positive clones” on the basis of phenotype (e.g. enzymatic activity), heterologous complementation and modulated detection by reporter genes [24]. As such, the approach does not depend on the availability of prior sequence information to detect enzymes and it therefore offers great potential to discover genetic novelty. Using this approach, searches for (hemi)cellulases have already been made in microbiomes from decaying wood, compost, rumen and soil [25–28]. However, only few studies have explored the enzymatic potential of microbial enrichments [29, 30]. It is important to notice that functional screenings come with a possible caveat, which relates to the fact that the expression conditions in the heterologous host used need to match the requirements of the insert. Due to this and other caveats (e.g. improper codon usage and/or promoter recognition, inclusion body formation, toxicity of the gene product or inability of the host to induce the gene expression), the frequency with which positive clones are uncovered may be very low [31]. In attempts to overcome such low hit rates, some studies have applied “biased” (e.g. substrate-enriched environment) samples, coupled to the use of highly sensitive chromogenic substrates (e.g. 5-bromo-3-indolyl-beta-D-xylopyranoside) [32]. Other studies have used plasmid vectors with dual-orientation promoters to obtain more positive clones [33]. The commonly-used substrates for screening for (hemi)cellulose-degrading enzymes include azo-dyed and azurine cross-linked polysaccharides (e.g. AZCL-HE-cellulose, AZCL-xylan or AZCL-beta-glucan), para-nitrophenyl glycosides (e.g. pNP-beta-D-cellobioside, pNP-alpha-galactopyranoside or pNP-alpha-L-arabinofuranoside), carboxymethylcellulose and rimazol brilliant blue dyed-xylan. However, multiple chromogenic substrates as proxies for functional screening for (hemi)cellulases have been underexplored, although, recently, these types of approaches were catalogued as highly interesting [34].

In this study, phylogenetically stable wheat straw-degrading microbial consortia [19–35] served as the sources for two fosmid-based metagenomic libraries. These libraries were subjected to expanded functional screens by a multi-substrate approach using six chromogenic compounds (indolyl-monosaccharides). Sequencing of the inserts of seven selected positive clones indicated the presence of a suite of novel genes encoding proteins of distinct glycosyl hydrolase (GH) families, which were flanked mostly by CBMs and ABC transporters. Thus, we present an effective strategy for exploration of fosmid libraries for (hemi)cellulases, revealing hit rates higher than those reported in previous studies. Subsequent functional analyses unveiled genes encoding putative novel thermo-alkaline-tolerant enzymes, which opens the way for future biotechnological applications (e.g. biorefining or bleaching pulp).

## Results

### Construction and functional screening of two fosmid metagenomic libraries

**Fig. 1:**
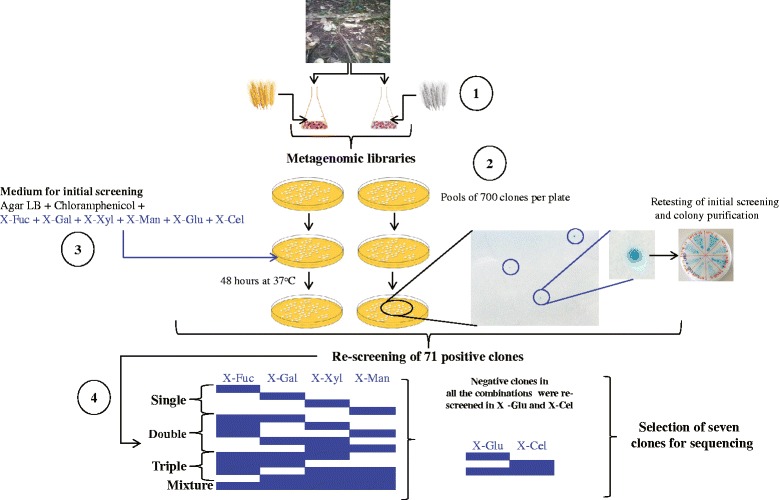
Schematic representation of the methodology used to screen for (hemi) cellulases in fosmid-based metagenomic libraries. **1)** Biased communities (e.g. soil-derived microbial consortia cultivated on wheat straw) are at the basis of enhanced hit rates. **2)** Screening of fosmid pools (700 fosmids per pool) allow high-throughput analyses. **3)** Screening on substrate mixtures accelerate the analyses. **4)** Re-screening of positive clones in single, double and further mixed combinations of substrates enable the detection of specific activities

Two metagenomic libraries were produced in fosmids, one from pooled raw wheat straw (RWS) consortial DNA (~70,000 clones) and another one from torrified wheat straw (TWS; ~70,000 clones). Each library contained clones with inserts of ~35 kb average size, yielding approximately 2.4 Gb of total cloned genomic DNA per library. In order to screen for (hemi)cellulose-degrading enzymes, about 22,000 clones per library were subjected to activity screens on LB agar supplemented with mixtures of six chromogenic substrates (Table 1; Fig. 1). These screens yielded a total of 71 positive hits, being 50 from RWS and 21 from TWS. This corresponded to, respectively, 1 hit per 440 screened clones (RWS), and 1 hit per 1,047 screened clones (TWS).

**Table 1:** Chromogenic substrates used in this study

Activity on^a^	Type of enzymes detected	Substrate (indolyl-monosaccharide)	Abbreviation	Concentration into the plate	Supplier
Hemicellulose	alpha-fucosidases	5-bromo-4-chloro-3-indolyl-α-L-fucopyranoside	X-Fuc	Each 40 μg/ml	Biosynth AG, Switzerland
	beta-galactosidases	5-bromo-4-chloro-indolyl-β-D-galactopyranoside	X-Gal		
	beta-xylosidases	5-bromo-4-chloro-3-indolyl-β-D-xylopyranoside	X-Xyl		
	alpha-mannosidases	5-bromo-4-chloro-3-indolyl α-D-mannopyranoside	X-Man		
Cellulose and Starch	beta-glucanases	5-bromo-4-chloro-3-indolyl-β-D-cellobioside	X-Cel		
	alpha-glucosidases	5-bromo-4-chloro-3-indolyl-α-D-glucopyranoside	X-Glu		

^a^Activity was predicted based on the linked monosaccharide

The 71 positive fosmid clones were restreaked to purity and then retested for activity to confirm the initial screening results. They were then subjected to additional plate screens with the hemicellulose-mimicking substrates X-Fuc, X-Gal, X-Xyl and X-Man, including single-, double- and mixed-substrate plates. The 71 positive clones showed activity in the latter assay (mixed-substrate plate), confirming the initial data. Clones that showed consistent activities on the mixtures of six substrates but no activity on the aforementioned hemicellulose-mimicking substrates were then tested for their abilities to degrade cellulose or starch using X-Cel and X-Glu (single and double) (Fig. 1). After removal of clones with questionable activity (e.g. faint-blue colonies with white background), the remaining 52 clones showed consistent activities on at least one of the substrates tested. Specifically, 20 clones showed activity on X-Gal (18 from RWS - hit rate 1:1,157; 2 from TWS - hit rate 1:11,000), 9 on X-Xyl (all from TWS – hit rate 1:2,444) and 15 on X-Glu (all from RWS – hit rate 1:1,466) (as singletons). Remarkably, eight were positive only on the mixture of six substrates.

For each fosmid clone type in our library, as determined by activity, 2–4 clones were selected for genetic analysis. This selection was also guided by the clones' origin, i.e. RWS or TWS. Restriction with *EcoR*1 revealed, for all tested fosmids, the presence of insert sizes of approximately 28 to 35 kb. It also allowed the detection of duplicates, for dereplication of the fosmid set. The, thus selected, final set of (seven) fosmids consisted of two clones that were positive for X-Gal (T17-2 and NT2-2), one for X-Xyl (T5-5), two for X-Glu (NT18-17 and NT18-21), and two with activity on multiple mixed substrates (10BT and T4-1) (Table 2).

**Table 2:** Selected fosmids for insert sequencing and annotation of genes

				Number of genes based on RAST platform and CAZy database annotation								
Fosmid ID	Positive on	# Contigs (total length)	% GC	GHs	CBMs	AAs	GTs	CEs	ABC	H/U	Others	Total
NT2-2	X-Gal	2 (31.21 Kb)	60.1	2	2	1	2	0	3	2	14	26
T5-5	X-Xyl	5 (31.63 Kb)	51.9	4	2	0	0	0	3	8	13	30
NT18-17	X-Glu	2 (33.7 Kb)	63.5	4	0	1	1	1	10	4	14	35
T4-1	Mixed^a^	2 (34.84 Kb)	57.2	2	3	1	0	0	3	5	17	31
10BT	Mixed^a^	2 (31.87 Kb)	54.6	2	0	2	1	0	1	12	25	43
NT18-21	X-Glu	1 (29.89 Kb)	55.0	0	1	0	0	0	3	2	21	27
T17-2	X-Gal	1 (23.57 Kb)	54.8	4	1	0	2	0	0	3	9	19

^a^positive fosmids on the mixture of six chromogenic substrates, as in Table 1

*GHs*, glycosyl hydrolases, *CBMs,* carbohydrate binding modules, *AAs,* auxiliary activities, *GTs*, Glycosyl transferases,*CEs*, carbohydrate esterases *ABC,* ABC transporters, *H/U,* Hypothetical and unknown genes

### Analysis of fosmid insert sequences and detection of carbohydrate-active enzymes

**Fig. 2:**
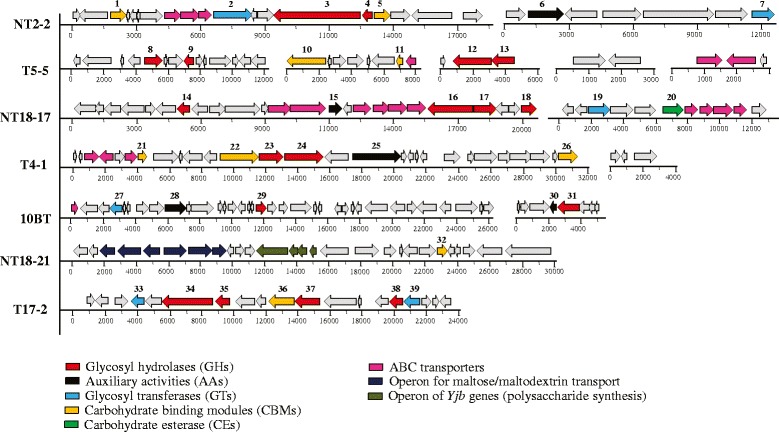
Graphical representation of the genes detected and annotated in seven fosmid inserts selected by the multi-substrate screening approach

The seven selected clones (NT2-2, T5-5, NT18-17, T4-1, 10BT, NT18-21 and T17-2), were subjected to full insert sequencing. Final assembly of the inserts revealed a total of 15 contigs, of sizes between 3.0 and 35 kb. Thus, some inserts had more than one contig, indicating the existence of regions with too low coverage. Per clone, the contigs were considered to be of sufficient representation to allow further analyses (Fig. 2). All contigs were then screened for the presence of open reading frames (ORFs) on the basis of the presence of start and stop codons (automatic annotation from the RAST server, followed by manual validation). In addition, the identified genes were, gene-by-gene, subjected to BLAST-based analyses, comparing against the NCBI and carbohydrate-active enzyme (CAZy) databases. Overall, we detected 18 promising ORFs encoding proteins from 12 different GH families amongst a total of 211 ORFs. The G + C contents of the inserts ranged from 54.6 to 63.5 %. The complete annotation of each of the seven fosmid inserts is presented in the supplementary files. A brief description of each insert is listed below (Tables 2 and 3).

**Table 3:** Carbohydrate-active genes detected in each fosmid insert

Fosmid ID	ID gene (Fig. 2)	CAZy Family ^a^	Size in amino acids (kDa ^b^)	Annotation based on RAST platform (EC number)	Probable protein from (identity / coverage)
NT2-2	1	CBM26	236	GCN5-related N-acetyltransferase	*Klebsiella oxytoca* (73 % / 83 %)
	2	GT2	521	Uncharacterized protein YlaB	*Enterobacter mori* (58 % / 74 %)
	3^c^	GH2	1028 (116)	Beta-galactosidase (EC 3.2.1.23)	*Enterobacter hormaechei* (74 % / 81 %)
	4	GH53	119	Transcriptional repressor of the lac operon	*Enterobacter hormaechei* (59 % / 75 %)
	5	CBM48	301	Carboxyl-terminal protease (EC 3.4.21.102)	*Pseudomonas putida* (99 % /100 %)
	6	AA3	565	Choline dehydrogenase (EC 1.1.99.1)	*Pseudomonas putida* (99 % / 99 %)
	7	GT90	144	Thioredoxin	*Pseudomonas putida* (95 % / 99 %)
T5-5	8	GH32	310	6-Phosphofructokinase class II (EC 2.7.1.11)	*Klebsiella oxytoca* (92 % / 97 %)
	9	GH13	241	Ferric siderophore transport system, periplasmic binding protein TonB	*Klebsiella pneumoniae* (75 % / 84 %)
	10	CBM20	792	Phosphoenolpyruvate synthase (EC 2.7.9.2)	*Klebsiella oxytoca* (98 % / 99 %)
	11	CBM50	154	Probable lipoprotein NlpC precursor	*Klebsiella oxytoca* (98 % /99 %)
	12^c^	GH3	789 (86)	Beta-xylosidase (EC 3.2.1.37)	*Enterobacter mori* (84 % /91 %)
	13	GH17	465	Xyloside transporter XynT	*Enterobacter cloacae* (82 % / 90 %)
NT18-17	14^c^	GH27	218 (22)	Aquaporin Z	*Hyphomonas neptunium* (65 % / 80 %)
	15	AA6	195	NAD(P)H oxidoreductase	*Rhizobium etli* (48 % / 64 %)
	16	GH20	642	Beta-hexosaminidase (EC 3.2.1.52)	*Rhizobium leguminosarum* 3841 (50 % / 64 %)
	17^c^	GH58	356 (38)	Hypothetical protein	*Hyphomicrobium denitrificans* (56 % / 71 %)
	18	GH109	231	Dehydrogenase	*Rhizobium leguminosarum* (58 % / 76 %)
	19	GT2	442	Omega-amino acid-pyruvate aminotransferase (EC 2.6.1.18)	*Mesorhizobium opportunistum* WSM207 (82 % / 90 %)
	20	CE9	483	Dihydropyrimidinase (EC 3.5.2.2)	*Rhizobium leguminosarum* (80 % / 90 %)
T4-1	21	CBM50	154	Probable lipoprotein NlpC precursor	*Klebsiella oxytoca* (92 % / 99 %)
	22	CBM20	792	Phosphoenolpyruvate synthase (EC 2.7.9.2)	*Klebsiella oxytoca* (98 % / 99 %)
	23^c^	GH17	465 (52)	Xyloside transporter XynT	*Enterobacter cloacae* (82 % / 90 %)
	24^c^	GH3	789 (86)	Beta-xylosidase (EC 3.2.1.37)	*Enterobacter mori* (83 % / 90 %)
	25	AA4/AA7	1000	Glycolate dehydrogenase (EC 1.1.99.14), subunit GlcD	*Klebsiella oxytoca* (95 % / 98 %)
	26	CBM50	332	L,D-transpeptidase YnhG	*Klebsiella oxytoca* (85 % / 93 %)
10BT	27	GT4	239	Thioesterase involved in non-ribosomal peptide biosynthesis	*Pseudomonas putida* (90 % / 93 %)
	28	AA3	413	Sarcosine oxidase beta subunit (EC 1.5.3.1)	*Pseudomonas putida* (98 % / 99 %)
	29^c^	GH39	192 (21)	Transcriptional regulator, AraC family	*Klebsiella oxytoca* (76 % / 87 %)
	30	AA5	127	Integral membrane protein YfiB	*Enterobacter cancerogenus* (90 % / 94 %)
	31^c^	GH53	406 (46)	Inner membrane protein YfiN	*Enterobacter cloacae* (79 % / 88 %)
NT18-21	32	CBM50	396	Shikimate 5-dehydrogenase I gamma (EC 1.1.1.25)	*Klebsiella oxytoca* (83 % / 90 %)
T17-2	33	GT8	207	Galactoside O-acetyltransferase (EC 2.3.1.18)	*Escherichia coli* (81 % / 93 %)
	34^c^	GH2	1027 (116)	Beta-galactosidase (EC 3.2.1.23)	*Citrobacter freundii* (87 % / 92 %)
	35	GH53	360	Transcriptional repressor of the lac operon	*Citrobacter koseri* (89 % / 94 %)
	36	CBM51	581	Choline-sulfatase (EC 3.1.6.6)	*Klebsiella oxytoca* (94 % / 97 %)
	37	GH32	471	PTS system, sucrose-specific IIB component	*Klebsiella oxytoca* (94 % / 97 %)
	38	GH65/GT5	266	Cof, detected in genetic screen for thiamin metabolic genes	*Klebsiella oxytoca* (97 % / 98 %)
	39	GT4	330	Lysophospholipase L2 (EC 3.1.1.5)	*Klebsiella oxytoca* (94 % / 97 %)

^a^Annotation using the CAZymes Analysis Toolkit (CAT) platform

^b^Predictive molecular size in kDa

^c^correspond to genes predicted to be involved in the detected enzymatic activities

### Fosmid NT2-2

Two contigs represented the total 31.2 kb insert, encompassing 26 predicted ORFs. The sizes of the identified ORFs ranged from 123 to 3,309 bp. Gene annotation revealed the presence of two predicted genes encoding proteins of GH families GH2 and GH53. The GH2-encoding gene was annotated as a beta-galactosidase (~116 kDa) and could be correlated with the activity on X-gal. Flanking this gene, genes predicted to encode two CBMs (CBM48 and CBM26), two glycosyl transferases (GTs) (GT2 and GT90) and an operon containing three methionine ABC transporter genes were found (Additional file 1).

### Fosmid T5-5

Thirty predicted ORFs, with sizes ranging from 138 to 2,379 bp, were present in the 31.6 kb insert (composed of five contigs). A gene predicted to encode a 789-amino acid protein (~86 kDa) was annotated as a gene for beta-xylosidase. This protein could be involved in the detected enzymatic activity on X-Xyl. Flanking this gene, three ABC transporters, a xyloside transporter (*Xyn*T – annotated as GH17 by CAZy) and a CBM50 gene (annotated as a lipoprotein by RAST) were found. In addition, predicted genes for carbohydrate-active enzymes of families CBM20, GH13 and GH32 were detected (Additional file 2).

### Fosmid NT18-17

Thirty-five ORFs were identified in the 33.7 kb insert (two contigs). The insert showed a high G + C content, i.e. 63.5 %. Four GH-encoding genes were detected, which fell in the GH27, GH20, GH58 and GH109 families. Of these, two predicted proteins might be linked to the activity on X-Glu (GH58-hypothetical protein or GH27-aquaporin). In addition, ten predicted ABC transporter genes were detected (Additional file 3).

### Fosmid T4-1

This contiguous insert (31.4 kb) consisted of 31 ORFs, of sizes between 189 and 3,003 bp. Two genes with predicted GH activity were found. These might be involved in the enzymatic activities detected on mixed substrates, i.e. a GH3 family gene (encoding a beta-xylosidase) and a GH17 family one (annotated as a xyloside transporter; *Xyn*T). In addition, three genes predicted to encode CBMs were found in this insert (two CBM50 and one CBM20). The remainder encompassed either hypothetical and/or uncharacterized genes (five ORFs) or genes encoding different functions (seventeen ORFs) (Additional file 4).

### Fosmid 10BT

Fourty-three ORFs were identified in the 31.8 kb insert of fosmid 10BT (two contigs). Consistent with the annotation, genes predicted to encode two GHs (i.e. GH39 and GH53), two auxiliary activities (AAs; AA5 and AA3), one GT (GT4) and one ABC transporter were identified. Of these, the newly discovered GH39 (beta-xylosidase) and GH53 (endo-beta-1,4-galactanase) genes could be related to the activities measured with the mixture of substrates. Interestingly, these genes were annotated -by RAST- as a transcriptional regulator (AraC family) and an inner membrane protein, *Yfi*N, respectively (Additional file 5).

### Fosmid NT18-21

Fosmid NT18-21 contained a 29.8 kb insert within a single contig. Although this insert contained a total of 27 predicted ORFs, CAZy annotation predicted only one gene with carbohydrate activity, i.e. one encoding CBM50 (identified by RAST as "shikimate 5-dehydrogenase I gamma"). Furthermore, two operons were detected that might relate to the activity, one of them encompassing predicted genes for maltose/maltodextrin transporters and the second one presumed polysaccharide synthesis genes (*Yjb*H-*Yjb*G-*Yjb*F-*Yjb*E). The latter operon was flanked by a gene for glucose-6-phosphate isomerase and one for an aspartokinase, which are both involved in sugar metabolism (Additional file 6).

### Fosmid T17-2

Nineteen ORFs were identified within the 23.5 kb insert of fosmid T17-2 (one contig), which had a G + C content of 54.8 %. The ORFs had a size range between 117 and 3,084 bp. One ORF, encoding a 1,027-amino acid protein, was identified as a gene for beta-galactosidase (GH2 family), suggesting it was responsible for the activity of the fosmid on X-gal. In addition, we identified genes for the transcriptional repressor of the lac operon and "PTS system sucrose-specific IIB component", that were identified by CAZy as a GH53 and a GH32 family proteins, respectively. Two genes encoding GTs (GT4 and GT8), and one gene for CBM51 were also identified (Additional file 7).

### Tracking the microbial sources of the fosmid inserts

To identify the potential microbial source of each metagenomic insert, the predicted amino acid sequences per gene per contig were BLAST-compared to the NCBI database. In addition, such BLAST results were analyzed by the Lowest Common Ancestor (LCA) algorithm in MEGAN v5 (Additional files). Thus, 14 predicted protein sequences from the NT2-2 insert were affiliated to proteins of members of the Enterobacteriaceae, notably *Klebsiella oxytoca* and *Enterobacter* sp. However, another 11 predicted proteins from this insert were affiliated, based on the 50 “best” BLAST hits, to those from *Pseudomonas putida*. In the fosmid T5-5 insert, 27 predicted proteins were mainly related to *Klebsiella oxytoca* –derived proteins. The insert of fosmid NT18-17 showed a complexity of genes that were affiliated to different genera (e.g. *Pelagibacterium*, *Rhizobium* and *Mesorhizobium*). These genera all belong to the Rhizobiales, suggesting an organism from this group as the most likely source. In both fosmids T4-1 and NT18-21, virtually all predicted proteins (approximately 96 %) were affiliated with proteins from members of the Enterobacteriaceae. Closer (manual) screening of the data indicated that insert T4-1 might come from a *Klebsiella oxytoca* -like organism, whereas insert NT18-21 might originate from an organism affiliated with either *Citrobacter*, *Klebsiella* or *Salmonella*. A similar observation was made for the fosmid T17-2 insert. In the case of fosmid 10BT, eleven ORFs yielded predicted proteins that resembled those of *Pseudomonas putida* –like organisms (coverage and identity of > 90 %), whereas the remainder of the predicted proteins were more related to those from enteric species (e.g. mostly *Klebsiella oxytoca* –like). This was similar to what was shown for the NT2-2 insert.

### Functional analyses: beta-galactosidase, beta-xylanase and alpha-glucosidase activities

**Fig. 3:**
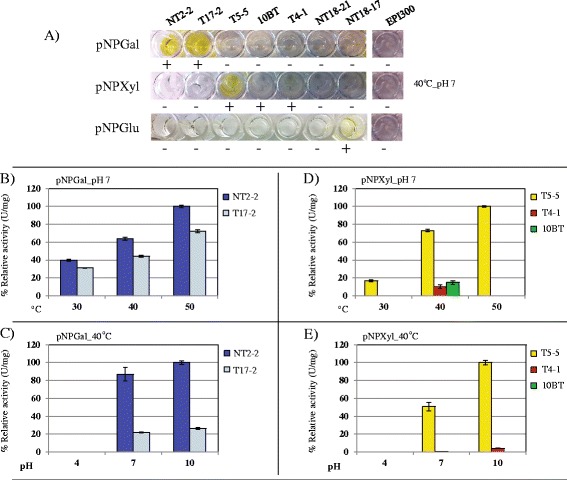
Functional analyses (relative enzymatic activities, expressed in U/mg) based on total proteins extracted from selected positive fosmid clones. **a** Characterization of the fosmid clones using three pNP-substrates (pNPGal, pNPXyl and pNPGlu). **b** and **c** Effect of temperature and pH on the activity of clones NT2-2 and T17-2 with pNPGal. **d** and **e** Effect of temperature and pH on the activity of clones T5-5, T4-1 and 10BT with pNPXyl

Based on the initially-detected activities of the fosmid clones, we selected three commercially available substrates, i.e. para-nitrophenyl-beta-D-galactopyranoside (pNPGal), para-nitrophenyl-beta-D-xylanopyranoside (pNPXyl) and para-nitrophenyl-alpha-D-glucopyranoside (pNPGlu), in order to quantify the activities (using total protein extracts) at different temperatures and pH values. Clones NT2-2 and T17-2 were positive on pNPGal, confirming the initial screening data, while clones T5-5, T4-1 and 10BT were positive on pNPXyl. In addition, clone NT18-17 showed activity on pNPGlu (Fig. 3a). Clones NT2-2 and T5-5 showed elevated levels of enzymatic activity and were therefore chosen for further assays (Fig. 3b,d). Total protein extracts produced from the fosmid-less host (*E. coli* EPI 300) did not show any activity on the selected pNP substrates, confirming that the activities came from the metagenomic inserts.

**Fig. 4:**
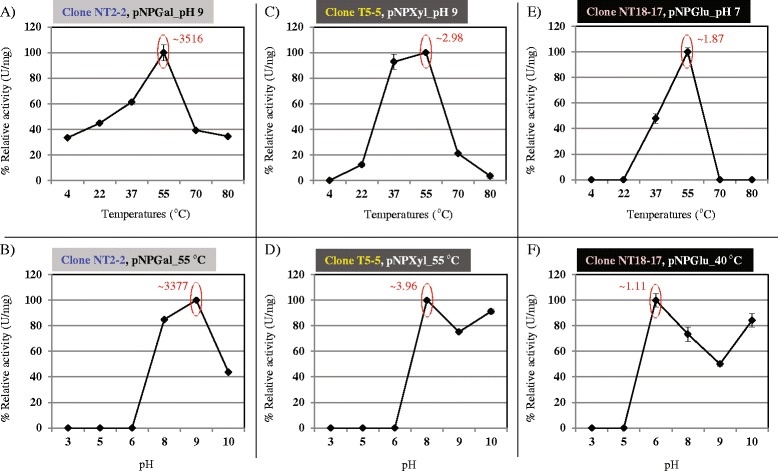
Relative enzymatic activities of the total proteins produced from the fosmid clones NT2-2, T5-5 and NT18-17 using pNPGal, pNPXyl and pNPGlu, respectively, at different temperatures and pH values. **a** and **b** Effect of temperatures and pH on the activity of fosmid clone NT2-2. **c** and **d** Effect of temperatures and pH values on the activity of the fosmid clone T5-5. **e** and **f** Effect of temperature and pH on the activity of fosmid clone NT18-17

For clone NT2-2, activity on pNPGal was maximal at 55 °C and pH 9.0 (between 3,516 ± 219.02 U/mg and 3,377 ± 47.19 U/mg ), with 34 % and 44 % of activity remaining at 80 °C and pH 10.0, respectively. Activity was not detected below pH 6.0 (Fig. 4a,b). The activity of clone T5-5 on pNPXyl was maximal at 55 °C, with values of around 93, 21 and 12 % of this maximum at 37, 70 and 22 °C, respectively. The highest xylanolytic activity for clone T5-5 was obtained at 55 °C and pH 8.0 (3.96 ± 0.04 U/mg ), with ~91 % of activity remaining at pH 10.0. The enzyme produced by T5-5 was likely alkaliphilic. Similar to clone NT2-2, T5-5 activity was not detected at pH values below 6.0 (Fig. 4c,d). Finally, clone NT18-17 showed maximum activity at pH 7.0 and 55 °C (1.87 ± 0.06 U/mg ), with about 0.94 U/mg at 40 °C and pH 10.0. This suggested this enzyme was quite tolerant to alkaline conditions (Fig. 4e,f).

### Zymograms

**Fig. 5:**
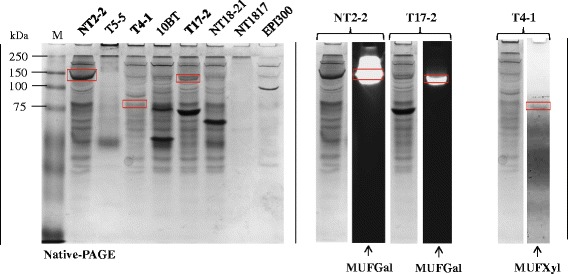
Native polyacrylamide gel electrophoresis (Native-PAGE) and zymogram analysis. Left: Native polyacrylamide gel of the total proteins from the positive fosmid clones. Right: Positive fosmid clones that showed bands (red square) with activity on the zymogram assay using MUFGal (MUF-beta-D-galactopyranoside) and MUFXyl (MUF-beta-D-xylopyranoside) as substrates

Native polyacrylamide gels showed that crude protein extracts from the seven fosmid clones had band patterns different from those of the *E. coli* EPI300 host. Moreover, none of the bands produced from the *E. coli* EPI300 host were positive with MUFGal (MUF-beta-D-galactopyranoside) and MUFXyl (MUF-beta-D-xylopyranoside) (used as substrates), confirming that any activities measured came from the metagenomic inserts. Clones NT2-2 and T17-2 both showed a band of >100 kDa with high activity on MUFGal (Fig. 5). Given their estimated sizes, these bands likely represent proteins encoded by genes 3 and 34 (Fig. 2; both beta-galactosidase encoding genes). Clone T4-1 showed a band of 75–100 kDa size, with xylosidase activity, which is consistent with the initial finding of activity on pNPXyl. This band likely corresponds to a protein encoded by gene 24 (Table 3), predicted to be a beta-xylosidase of ~86 kDa. Clones T5-5 and 10BT, positive with pNPXyl, did not show any bands with activity on the zymograms using MUFXyl.

## Discussion

In this study, two wheat straw-degrading microbial consortia, RWS and TWS, were successfully subjected to metagenomic library constructions using fosmids, yielding 2.4 Gb of genomic information per library. Taking an estimated average bacterial genome size in our microbial consortia of 4 Mb and considering these were strongly dominated by bacteria, we thus cloned the equivalent of roughly 600 bacterial genomes. Previous data on the two consortia [35] revealed the presence of ~100 (RWS) and ~50 (TWS) dominant bacterial types, in relative abundances within one log unit, giving a coverage of around six-fold for RWS and twelve-fold for TWS. Hence, a back-of-the-envelope calculation revealed that we basically covered the genes from most of the dominant bacterial members in the degrader consortia.

To detect (hemi)cellulolytic enzymes by functional screenings, two alternative strategies can be followed: 1) high-throughput detection in agar plates (mostly secreted enzymes) using hydrolysis of a chromogenic substrate as the criterion and 2) detection of enzyme activity in crude extracts after cell lysis. In either methodology, additional factors should be taken into account. These are the vector copy number, the need for induction of gene expression, secretion of the enzyme and recovery of the vector plasmid after expression [31, 36]. Here, we tested our fosmids by functional screenings for (hemi)cellulases initially using a mixture of (six) chromogenic compounds in agar plates. These substrates (indolyl-monosaccharides) can be internalized by *E. coli* and are thus readily available for hydrolysis by intracellularly-expressed exo-glycosidases. This is not the case for substrates such as oat spelt xylan or CMC, the hydrolysis of which relies on the release of fosmid-expressed enzymes, which probably only occurs after cell death and lysis [32]. The substrates were organic compounds, each consisting of a monosaccharide linked to a substituted indole moiety. The substrates yield insoluble blue compounds as a result of enzyme-catalyzed hydrolysis. For example, X-Xyl, when cleaved by beta-xylosidase, yields xylose and 5-bromo-4-chloro-3-hydroxyindole. The latter compound can spontaneously dimerize and is oxidized into 5,5′-dibromo-4,4′-dichloro-indigo, an intensely blue product which is insoluble. Taking into account the structure of these substrates, we hypothesized that the approach allows the screening for debranching enzymes that act in the external chains of sugars (in this case fucose, xylose, galactose, mannose and glucose) and that are linked to the backbone of the (hemi)cellulose structures. Indeed, our multi-substrate approach is also applicable in screens of metagenomic libraries for other classes of enzymes, as long as chromogenic substrates are available for that purpose. For example, to detect lipolytic activity, 5-(4-hydroxy-3,5-dimethoxyphenylmethylene)-2-thioxothia-zolidin-4-one-3-ethanoic acid (SRA)-propionate, SRA-butyrate, SRA-octanoate, SRA-decanoate, SRA-laurate and SRA-myristate can be employed [37].

Given our high hit rates, i.e.1:440 in RWS and 1:1,047 in TWS, the multi-substrate screening approach was superior to approaches reported in the recent literature (Table 4). On the other hand, in both libraries the hit rates for the individual enzymes were <1:2,500, except for X-Gal in RWS (1:11,000). Comparing 15 different metagenomic libraries and 19 single substrates, we inferred an average hit rate of (hemi)cellulolytic activities of ~ 1:7,300. However, some approaches showed hit rates of < 1:2,000. Additionally, low hit rates were found (in recent studies) using chromogenic substrates (e.g. X-Xyl) or azurine cross-linked polysaccharides. Interestingly, Zhao et al. [38], screening a BAC vector library produced from a cow rumen microbiome, reported a hit rate of 1:853 using xylan as the screening substrate. Nguyen et al. [39], screening their buffalo rumen metagenomic library, found hit rates of 1:108 and 1:2,500 using AZCL-HE-cellulose and AZCL-xylan, respectively. The authors suggested that the relatively high hit rate on AZCL-HE-cellulose can be attributed to the use of ENZhance cell permeabilizing reagent. These results emphasize the advantages of combining large-insert libraries (maximizing the probability of identifying gene clusters whose components perform complementary functions), enriched-function systems (such as the cow rumen) and reagents that enhance the host cell permeabilities, allowing the release of enzymes.

**Table 4:** Comparison of functional metagenomic approaches to screen for (hemi)cellulases

Microbial source (enrichment bred from)	Vector / Host	Screened clones	Substrates for (hemi)cellulases screening	Hit rate ^f^	Reference
Cow rumen	pCCl-BAC ^a^/ *E. coli* EPI300	15360	Xylan	1/853	Zhao et al. (2010) [38]
Termite gut	pCC1FOS ^b^ /*E. coli* EPI100	40000	5-Bromo-3-indolyl-beta-D-xylopyranoside	1/740	Bastien et al. (2013) [32]
		40000	5-bromo-4-chloro-3-indolyl-alpha-L-arabinofuranoside	1/1.212	
		40000	Oat AZCl-xylan	1/4.444	
Cow rumen	pCCFOS ^b^ / *E. coli* EPI300-T1	17000	pNP-beta-D-glucopyranoside + pNP-beta-D-cellobioside	1/5.666	Del Pozo et al. (2012) [16]
Buffalo rumen	pCCFOS1 ^b^ / EPI300-T1	10000	AZCL-HE-cellulose	1:108	Nguyen et al. (2012) [39]
		10000	AZCL-xylan	1:2.500	
Avicel- enrichment culture (soil)	pUC19 ^c^ / *E. coli* DH5α	57000	Rimazol brilliant blue dyed-CMC	1/7.125	Mori et al. (2014) [29]
Pulp- enrichment culture (soil)		63000	Rimazol brilliant blue dyed-xylan	1/63.000	
Ikaite tufa columns	pGNS-BAC ^a^ / *E.coli* DH10B	2843	5-bromo-4-chloro-3-indolyl-beta-d-galactopyranoside	1/1.421	Vester et al. (2014) [49]
Grassland soil	pCC1FOS ^b^ / *E. coli* EPI300	4600	Dye-labeled hydroxyethyl xylan	1/4.600	Nacke et al. (2012) [28]
		4600	Dye-labeled hydroxyethyl cellulose	1/2.300	
Human gut	pCC1FOS ^b^ / *E. coli* EPI300	156000	AZCL-xylan	1/4.588	Tasse et al. (2010) [50]
		156000	AZCL-beta-glucan	1/1.772	
		136000	AZCL-galactan	1/1.271	
Filter paper -enrichment culture (Earthworm)	pCCFOS ^b^ / *E. coli* EPI300	115000	pNP-beta-D-glucopyranoside	1/2.090	Beloqui et al. (2010) [30]
Compost from pig manure	pCC2FOS ^b^ / *E. coli* EPI300	12380	Xylan	1/2.476	Jeong et al. (2012) [51]
Forest soil	Lambda ZAP Express ^c^ / *E. coli* XL1 blue	10000	CMC	1/10.000	Wang et al. (2009) [52]
Elephant dung		10000	CMC	1/5.000	Wang et al. (2009) [52]
Cow rumen	pCCFOS ^b^ / *E. coli* EPI300	12288	pNP-alpha-galactopyranoside	1/12.288	Ferrer et al. (2012) [27]
		12288	pNP-alpha-L-arabinofuranoside	1/4.096	
		12288	CMC	1/6.144	
Alkaline-polluted soil	pGEM-3Zf ^c^ / *E. coli* DH5a	30000	Esculine	1/15.000	Jiang et al. (2011) [53]
Soil	pHBM803 ^c^ / *E. coli* XL10-Gold	24000	Remazol brilliant blue dyed-xylan	1/24.000	Hu et al. (2008) [54]
^13^C-cellulose-enriched DNA (soil) ^e^	pJC8 ^d^ / E. coli HB101	2876	CMC	1/360	Verastegui et al. (2014) [40]
Wheat straw- enrichment culture (soil)	pCC2FOS ^b^ / *E. coli* EPI300	22000	Mixture of 6 indolyl-monosaccharides	1/440	This study
Torrefied wheat straw- enrichment culture (soil)		22000	Mixture of 6 indolyl-monosaccharides	1/1,047	

^a^Bacterial artificial chromosome

^b^Fosmid

^c^Plasmid

^d^Cosmid

^e^High-molecular weight DNA from the ^13^C-cellulose-enriched SIP incubations for the three soils

^f^Hit rate was determined based on the number positives clones in the initial screening / total of screened clones

*AZCL* (azurine cross-linked)

*CMC* (carboxymethylcellulose)

*pNP* (para-nitrophenyl)

Here, “biased” communities were produced on wheat straw as the carbon source and energy, which is thought to raise the relative abundance of target genes in the consortium and thus in the fosmid clones. However, Mori et al. [29], using a pUC19 library produced from pulp enrichments, obtained a hit rate of only 1:63,000 with rimazol brilliant blue dyed-Xylan. Beloqui et al. [30] reported a hit rate of 1:2,090 in a library prepared from filter paper -enrichments inoculated from earthworm gut extract, using as the screening substrate pNP-beta-D-glucopyranoside. Another interesting approach that potentially leads to highly efficient discovery of GH activities is the construction of metagenomic libraries prepared from DNA selected following stable isotope probing (DNA-SIP) using multiple labeled plant-derived carbon substrates. For example, Verastegui et al. [40] showed a hit rate of 1:360 using DNA from ^13^C-cellulose-enriched incubations on the basis of CMC as the substrate (Table 4). Clearly, the rates of obtaining positive clones are related to the cloning vector used, the metagenome source, the screening technique (substrates and desired activity) and the host cells. On top of that, in many cases stochastic (chance) factors play a role as well, which may relate to the relatively low sample sizes [36].

In functional screening of metagenomic libraries, proper selection of the substrate is highly recommended. Initial selection of substrate-active clones with “general” substrates or mixtures of substrates followed by more specific ones may represent a desirable “layered” approach. Recently, a new generation of multi-colored chromogenic polysaccharide substrates has been developed [41]. These substrates can be used to screen for GH activities (in this case, focusing on endo-enzymes). They show versatility and are convenient for high-throughput analyses for first-level screenings. Additionally, substrates representing -at least partially- the complexity of plant cell walls were produced, enabling activity screens on “real-world” plant polysaccharides.

In our study, four fosmid inserts carried genes that could be directly linked to the enzymatic activities based on homologies to known enzymes and predicted and detected protein sizes. Thus, proteins of predicted sizes (1,027 and 1,028 amino acids, giving proteins of ~116 kDa) from fosmids NT2-2 and T17-2 (selected as positive on X-Gal) did transform pNPGal and MUFGal (zymogram). These were both annotated as beta-galactosidases of family GH2 (EC 3.2.1.23) (Table 3). Fosmids T5-5 and T4-1, positive on X-Xyl/pNPXyl and mixed chromogenic substrates, respectively, revealed the presence of genes predicted to produce proteins of 789 amino acids (~86 kDa), which were annotated as beta-xylosidases of family GH3 (EC 3.2.1.37). Interesting, fosmid T4-1 showed activity only on the substrate mixes, but not on single X-Xyl. This clone showed slight activity on pNPXyl (0.113 ± 0.002 U/mg  at 40 °C, pH 7.0) and revealed a protein of size between 75 to 100 kDa, which was likely encoded by gene 24 (Table 3). The protein was positive on the zymogram using MUFXyl as a substrate (Fig. 5). The expression of this GH3 family gene may be regulated by the presence of the other substrates. Based on this rationale, on X-Xyl alone its activity might not be detected, whereas the presence of other substrates might spur activity, similar to what may happen in nature. Such a finding opens up a new paradigm in the screening of active enzymes from metagenomic libraries. Interestingly, in fosmid T4-1 a predicted xyloside transporter gene (*Xyn*T) was detected, which matched the CAZy GH17 family. Proteins from this family can have glucan endo-1,3-beta-glucosidase activity, suggesting a possible involvement in activity on mixed substrates.

Although fosmid NT18-17 was positive on X-Glu and pNPGlu, we did not detect any gene related with its predicted alpha-glucosidase activity. However, the detected genes for family GH58 (endo-N-acetylneuraminidase) and family GH27 (alpha-galactosidase) proteins might encode the activity (see Additional file 8 for a summary of activities associated with CAZy enzyme families described in this study). Similarly, fosmid NT18-21 showed alpha-glucosidase activity, with no GH family genes being detected in the insert. In this fosmid, genes for maltose/maltodextrin transporters were found. Maltose (an alpha 1–4 linked glucose dimer) resembles a cellulose dimer (albeit beta 1–4 linked). Maltose is released from starch by amylose/amylopectin-degrading activity (e.g. GH13- alpha-amylase, pullulanase or alpha-glucosidase). We surmised that starch that was initially present in the wheat straw used for consortium breeding incited the selection of such systems. The chromogenic substrates, e.g. X-Glu, used in this study were surmised to report alpha-glucosidase activities, but genes for such enzymes were not detected. Possibly, gene 25 (annotated as an alpha-aspartyl dipeptidase; EC 3.4.13.21) or gene 24 (hypothetical protein) were responsible for the activity (Additional file 6).

Fosmid clone 10BT showed consistent activity only on mixed substrates. In addition, 10BT showed activity on pNPXyl, much below that shown by clone T5-5 (~14,8 % of relative activity at 40 °C, pH 7.0; Fig. 3d). The finding of two genes producing proteins related to GH39 and GH53 families was revealing. Interestingly, family GH39 proteins have been linked to beta-xylosidase and alpha-L-iduronidase activities. Moreover, GH53 family proteins can have beta-1,4-galactanase activity (EC 3.2.1.89). The latter is possibly linked to the degradation of galactans and arabinogalactans, both integral parts of the pectin component of plant cell walls [42]. Interestingly, Jiménez et al. [43] recently found a novel cold-tolerant esterase, which had originally been annotated as a MarR family transcriptional regulator. Thus, we surmised that the gene that was originally predicted to encode an AraC transcriptional regulator may be responsible for the activity on pNPXyl. Similar to clone T4-1, this clone could require the presence of other types of substrates to enable detection of its full plethora of activities. However, given that we still don’t know the mechanism involved, further studies are required, for example subcloning, transposon mutagenesis and detection of activities on different substrates.

The high activity of fosmid NT2-2 compared to that of clones T5-5 and NT18-17 suggested a raised expression of the gene encoding beta-galactosidase (~116 kDa, as evident by the zymogram; Fig. 5). Interestingly, the high activities of clones NT2-2 and T5-5 at 55 °C and pH 10.0 pointed to their potential usefulness in pulp bleaching processes. The novelty attributed to these genes (3 and 12) was based on the low amino acid identities (less than 84 %) and coverage values (less than 91 %) versus the best hits in the NCBI database (Table 3). The functional analysis done by us directly from the metagenomic clones indicated substrate specificities and temperature/pH optima, and constitutes an easy way to select clones useful for biotechnology applications. In addition, subcloning, overexpression, induction and subsequent protein purification are labor-intensive and not always successful (e.g. due to low solubility of the enzyme).

The leading industrial source of cellulase cocktails used for plant biomass biodegradation purposes is *Trichoderma reesei*. Several strains exist and their secretomes have been widely used to develop new commercial cocktails. However, *T. reesei* secretomes are dominated by endoglucanases and it usually produces low quantities of xylanases, arabinofuranosidases, galactosidases and beta-glucosidases. Hence, addition of exogenous enzymes to the secreted fraction could improve the hydrolytic efficiency [44]. Based on this premise, the enzymes detected in clones NT2-2, T5-5 and NT18-17 might serve as components of new (hemi)cellulolytic cocktails. These may be combined with the commercial cellulases to improve plant biomass degradation for second-generation biofuel production. Additionally, thermo-alkaliphilic xylosidases are valuable with respect to their usefulness in pulp bleaching processes [13, 14].

In a previous study [45], Bacteroidetes-related genes for hemicellulases were found to be prominent amongst the dominant enzymes, whereas *Klebsiella*-related ones were less abundant. Both groups of organisms are key dominant types in our bacterial consortia bred on wheat straw. In the current study, evidence was found for the contention that *Klebsiella-*related organisms were at the basis of most cloned genes for biodegradative enzymes. The taxonomic closeness between this putative source organism (*Klebsiella*) and the heterologous host (*E. coli*) used, versus the remoteness in the case of Bacteroidetes, may have been a key factor explaining this finding. Unfortunately, the current study did not detect fosmids with activities on X-Fuc and X-Man. Such activities might be mostly associated with members of the Bacteroidetes (e.g. *Sphingobacterium*), as recently indicated by Jiménez et al. [45]. Finally, the differential association of fosmid NT2-2 and 10BT genes with *Pseudomonas putida* versus *Klebsiella* sp. was remarkable. IS-elements indicative of horizontal gene transfer were not detected, suggesting these fosmids might originate from fusions of two regions originating from different parental organisms. Alternatively, the insert may have come from a new Gammaproteobacteria species.

## Conclusions

Here, we propose a multi-substrate screening approach as a sound strategy that allows to detect multiple activities in a single initial assay. This methodology is less time-consuming than single-substrate approaches and can even be applied in high-throughput set-ups, as in agar plates. The strategy yielded high hit rates of genes for relevant enzymes compared with recent relevant literature data. Based on this methodology, we retrieved fosmids with beta-galactosidase, beta-xylosidase and alpha-glucosidase activities, whereas other fosmids showed activity only in the presence of mixed chromogenic substrates. Two fosmids, NT2-2 (GH2- beta-galactosidase) and T5-5 (GH3- beta-xylosidase), showed enzymatic activities at high temperatures and pH values, making these clones interesting sources for future biotechnological applications.

## Methods

### Metagenomic DNA extraction from lignocellulolytic microbial consortia

The lignocellulolytic microbial consortia examined here were primed with a forest soil derived microbial source. Briefly, the microbial cells were introduced into triplicate flasks containing 25 ml of mineral salt medium (MSM) with 1 % of *i*) “raw” wheat straw (RWS) and *ii*) heat-treated (240 °C, 1 h) wheat straw (TWS), after which flasks were incubated at 25 °C with shaking at 100 rpm. A sequential-batch approach was followed up to the 10^th^ transfer [19–35]. Then, DNA was extracted from the 10^th^ transfer microbial consortia, by using the UltraClean® Microbial DNA Isolation Kit (MoBio Laboratories Inc., USA). Three replicate crude DNA extracts were pooled for each fosmid library and concentrated up to 250 ng/μl using a Speedvac concentrator (Eppendorf, Hamburg, Germany).

### Construction of metagenomic libraries in fosmids

Metagenomic libraries were constructed using the CopyControl^TM^ HTP Fosmid Library Production Kit (Epicentre Biotechnologies, Madison, USA). Briefly, the metagenomic DNA was partially sheared by pipetting, to yield DNA fragments between 30 to 50 kb, after which it was 5’phosphorylated / blunt-ended. The DNA was then analyzed in 1 % low-melting-point agarose using a CHIEF-DR III pulsed field gel electrophoresis system (BioRad, Hercules, USA) at 14 °C with the following parameters: gradient 6 V/cm, included angle 120°, initial switch time 0.5 s, final switch time 8.5 s, linear ramping factor, 18 h. DNA fragments of approximately 30–40 Kb were excised from the gel and recovered using Zymoclean^TM^ Large Fragment DNA Recovery Kit (Zymo Research, Irvine, USA). The DNA was then ligated into vector pCC2FOS, packaged in phage particles and competent *E. coli* EPI300-T1^R^ cells were transformed with it. The *E. coli* cells were diluted 1:10^3^ and plated onto 1 % LB agar supplemented with 12.5 μg/ml chloramphenicol (LBA + Cm). Plates were incubated overnight at 37 °C, to produce 500 to 600 colonies per plate. The colonies of each plate were pooled in 1 ml of LB broth with 20 % of glycerol and stored as fosmid pools at −80 °C for further analysis.

### Screening for fosmid clones expressing (hemi)cellulolityc enzymes

Screening was done in three steps. First, fosmid pools stored at −80 °C were recovered in 100 μl LB broth at 37 °C for 1 h (shaking at 250 rpm) and serially diluted up to 1:10^5^. Then, each suspension (100 μl) was plated on LBA + Cm supplemented with a mix of each of the six chromogenic substrates (at 40 μg/ml) (Table 1). After incubation (48 h, 37 °C), dark blue colonies (due to hydrolysis of the chromogenic substrate) were selected, purified to obtain single colonies and retested. Secondly, selected clones were plated onto LBA + Cm supplemented with each of the specific hemicellulose-mimicking substrates, i.e. X-Fuc, X-Gal, X-Man and X-Xyl, in single, double, triple and quadruple combinations. Thirdly, clones that were positive in the first screening (on six substrates) and negative in the second screening (four substrates in different combinations) were further tested on X-Cel and X-Glu (single and double combinations) (Fig. 1).

### Extraction of DNA from selected fosmid clones

Selected positive clones were cultured in 4 ml of LB supplemented with 12.5 μl/ml chloramphenicol (LB + Cm) and incubated at 250 rpm for 8 h at 37 °C. After incubation, 25 μl was used to inoculate 25 ml of fresh LB + Cm. To increase the fosmid copy numbers, 50 μl of autoinduction solution (500X) (Epicentre Biotechnologies, Madison, USA) were added and flasks incubated (37 °C, shaking at 250 rpm). At OD_600_ of about 2–2.5, fosmid DNA was extracted from these cultures using the Gene Jet Plasmid Midi Preparation Kit (Thermo Scientific, Waltham, USA). DNA size and integrity were verified by running aliquots of the DNA on 1 % agarose gels and DNA concentration was measured by spectrophotometry (Nanodrop 2000; Thermo Scientific). The resulting fosmid DNA was digested with *EcoR*1 and the restriction patterns were analyzed on 0.8 % agarose gels. Band sizes were estimated by comparison to a standard DNA marker (GeneRuler^TM^ 1 Kb ladder, Thermo Scientific). The size of the insert of each fosmid was estimated by calculating the sum of the sizes of the individual *EcoR*1 generated bands minus 8,181 bp (fosmid backbone).

### Sequencing and gene annotation of fosmid inserts

The selected positive fosmid clones were sequenced using the Illumina HiSeq platform (2 X 100 bp). Sequences from the *E. coli* EPI300 genome as well as vector backbone sequences were removed. The resulting set of raw sequence data was quality-checked and further processed, with normalization and Velvet-based *de novo* assembly to generate contigs (Beckman Coulter Genomics, Danvers, USA). Contigs were selected from the data if the average coverage exceeded 200-fold, and final contigs were considered to be representative of the whole insert. ORFs were assigned to each of the contigs using the Rapid Annotation Subsystems Technology (RAST) server [46]. Subsequently, the ORFs were annotated in the CAZymes Analysis Toolkit (CAT) platform [47], using default parameters. Finally, all genes predicted in each fosmid insert were re-annotated and verified (in-house) using BLASTX searches against the NCBI database. The results from this analysis were loaded into MEGAN v5 software [48], after which they were classified taxonomically using the suggested parameters for the LCA algorithm (maximum number of matches per read 10; minimal support 5; minimal score 35; max expected 0.01; minimal complexity 0.3; and top percent 10). The nucleotide sequences of the contigs retrieved from the clones NT2-2, T5-5, NT18-17, T4-1, 10BT, NT18-21 and T17-2 were deposited in the GenBank database under the accession numbers KU505133-KU505147.

### Functional analyses – beta-galactosidase, beta-xylanase and alpha-glucosidase activity assays

Positive clones were grown (37 °C, shaking at 250 rpm) in 5 ml LB + Cm containing 10 μl of auto-induction solution (500X). At OD_600_ of 0.5, cells were harvested by centrifugation (10 min, 10,000 g). Proteins were extracted by adding 2 ml of lysis buffer (20 mM Tris–HCl pH 7.5, 100 mM NaCl, 1 mM EDTA, 0.1 % Triton, 5 mM CHAPS and a tablet of protease inhibitor -Roche- to 50 ml) to the pellet. Subsequently, the mixtures were sonicated on ice (6 s on, 15 s off, 30 cycles with amplitude of 10–15 microns). Protein concentration was determined by the Bradford method using bovine serum albumin as standard. The total protein fractions were recovered and tested for activity using pNPGal, pNPXyl and pNPGlu. The reaction mixture consisted of 0.3 ml of 10 mM pNPGal, pNPXyl or pNPGlu (diluted in 50 mM of Tris–HCl pH 7.0) and 0.3 mL of each clone protein fraction. The mixtures were incubated at 40 °C for 30 min to 2 h (depending on the quantity of proteins and activity) and the reactions were stopped on ice. Two negative controls were used for all assays: *i*) reaction mixture without substrate and *ii*) reaction mixture using the total protein fraction from the fosmid-less host *E. coli* EPI300. Enzymatic activities were determined from the measured absorbance units using a standard calibration curve. The amount of para-nitrophenol liberated was measured by absorbance at 410 nm. One unit (U) of enzyme activity was defined as the activity required for the formation of 1 μM of pNP per min under the above conditions (in this case mg of total protein). Optimum temperature was determined in the range of 4–80 °C using pNPGal, pNPXyl and pNPGlu (at pH 7.0 and 9.0). The pH optimum was determined in a pH range from 3.0 to 10.0 (at 40 °C and 55 °C) under standard conditions using the following buffers: 50 mM sodium citrate (pH 3.0 to 6.0), 50 mM Tris–HCl (pH 7.0–9.0) and 50 mM glycine-NaOH (pH 10.0).

### Protein electrophoresis and zymographic analyses

Zymograms were used to detect beta-galactosidase and beta-xylosidase activities on native polyacrylamide gels (4 % stacking, 10 % resolving gels) using 40 μg of total protein per sample. After running the gels at 4 °C, they were washed with water and then incubated with 5 mM of each substrate (MUFGal and MUFXyl) diluted in 0.1 M of Tris–HCl (pH 8.0) at 25 °C for 1 h. Following this, bands were visualized under UV light.

## Availability of supporting data

All the supporting data are included as additional files

## Additional files

Additional file 1:
**Complete gene annotation of fosmid insert NT2-2.** (XLS 41 kb) Additional file 2:
**Complete gene annotation of fosmid insert T5-5.** (XLS 42 kb) Additional file 3:
**Complete gene annotation of fosmid insert NT18-17.** (XLS 43 kb) Additional file 4:
**Complete gene annotation of fosmid insert T4-1.** (XLS 43 kb) Additional file 5:
**Complete gene annotation of fosmid insert 10BT.** (XLS 43 kb) Additional file 6:
**Complete gene annotation of fosmid insert NT18-21.** (XLS 39 kb) Additional file 7:
**Complete gene annotation of fosmid insert T17-2.** (XLS 36 kb) Additional file 8:
**Summary of activities associated with CAZy enzyme families described in this study.** (XLS 40 kb)

## Abbreviations

AAs: auxiliary activities
AZCL: Azurine cross-linked polysaccharides
CAZy: carbohydrate-active enzyme database
CBMs: carbohydrate-binding modules
CEs: carbohydrate esterases
CMC: Carboxymethyl cellulose
GHs: glycosyl hydrolases
GTs: glycosyl transferases
LCA: lowest common ancestor algorithm
MUF: 4-methylumbelliferyl
ORF: open reading frame
pNP: para-nitrophenyl
pNPGal: para-nitrophenyl beta-D-galactopyranoside
pNPGlu: para-nitrophenyl alpha-D-glucopyranoside
pNPXyl: para-nitrophenyl beta-D –xylanopyranoside
RAST: rapid Annotations using Subsystems Technology
X-Cel: 5-bromo-4-chloro-3-indolyl-β-D-cellobioside
X-Fuc: 5-bromo-4-chloro-3-indolyl-α-L-fucopyranoside
X-Gal: 5-bromo-4-chloro-indolyl-β-D-galactopyranoside
X-Glu: 5-bromo-4-chloro-3-indolyl-α-D-glucopyranoside
X-Man: 5-bromo-4-chloro-3-indolyl α-D-mannopyranoside
X-Xyl: 5-bromo-4-chloro-3-indolyl-β-D-xylopyranoside
